# Modified transcanal cochlear implantation in CHARGE syndrome: A case report

**DOI:** 10.1097/MD.0000000000018283

**Published:** 2019-12-16

**Authors:** Cheng-Yu Hsieh, Chuan-Hung Sun, Wei-Lin Lin, Giselle L. Gotamco, Chuan-Jen Hsu, Hung-Pin Wu

**Affiliations:** a Department of Otolaryngology, Head and Neck Surgery, Taichung Tzu Chi Hospital, Buddhist Tzu Chi Medical Foundation; b School of Medicine, Tzu Chi University, Hualien, Taiwan; c Section of Otorhinolaryngology - Head and Neck Surgery, Department of Surgery, Chinese General Hospital and Medical Center, Manila, Philippines.

**Keywords:** CHARGE syndrome, cochlear implantation, transcanal approach

## Abstract

**Rationale:**

Cochlear implantation (CI) in CHARGE syndrome is technically challenging because of the anatomical anomalies. This case aims to report a successful case of CI in CHARGE syndrome by using the modified transcanal approach with external auditory canal (EAC) obliteration.

**Patient concerns:**

The 3-year-old girl presented at the outpatient department with bilateral hearing loss and nasal obstruction since birth.

**Diagnosis:**

The patient had bilateral profound sensorineural hearing loss, patent ductus arteriosus, atresia of the choanae, middle and inner ear anomalies, and growth retardation, fulfilling the criteria for typical CHARGE syndrome. High resolution temporal bone computed tomography scan revealed a poorly developed mastoid cavity, cochlear dysplasia, hypoplastic semicircular canals, ossicular chain malformation, and sigmoid sinus engorgement. Magnetic resonance imaging revealed a narrow internal auditory canal and a hypoplastic cochlear nerve.

**Interventions:**

Modified transcanal approach with external auditory canal obliteration

**Outcomes:**

CI was successfully done and there are no intraoperative or postoperative complications occurred after 1 year of follow up.

**Lessons:**

The modified transcanal approach is a reasonable and safer option for CI in CHARGE syndrome

## 1 Introduction

CHARGE syndrome is characterized by multiple congenital anomalies that affect major organs, including the ears, eyes, and nose. It also impedes normal growth and development.^[1]^ In CHARGE syndrome with profound hearing loss, cochlear implantation (CI) may be the most promising option for successful hearing restoration.^[2,3]^

However, CI in CHARGE syndrome is technically challenging because of the presence of a poorly developed mastoid, ossicular chain malformation, aberrant facial nerve, hypoplastic semicircular canal, absent or covered round window, and dysplastic cochlea.^[4,5,6]^ Because of these anatomical anomalies, the conventional facial recess approach may not be the most appropriate surgical technique.

We report a successful CI technique in CHARGE syndrome by using the modified transcanal approach with external auditory canal (EAC) obliteration.

## 2 Case report

The 3-year-old girl had bilateral profound SNHL, patent ductus arteriosus, atresia of the choanae, middle and inner ear anomalies, and growth retardation fulfilling the criteria of typical CHARGE syndrome.^[7]^

**Figure 1:**
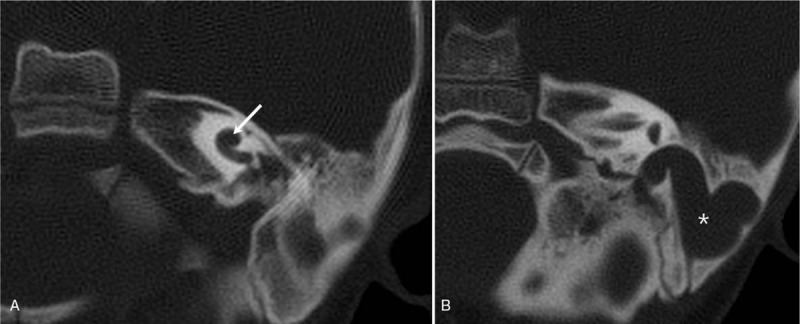
Radiographic finding (axial computed tomography) from the left ear of the patient demonstrating characteristics of CHARGE syndrome. A. the cochlear is dysplastic (arrow). B. the sigmoid sinus shows engorgement (asterisk).

Pure tone audiometry revealed bilateral profound sensorineural hearing loss. The aided threshold was approximately 60 dB. The auditory steady state response showed thresholds to be more than 110 dB bilaterally. The click auditory brainstem response (cABR) was 80 dB on the left ear and elicited no response on the right. High resolution temporal bone CT scan revealed a poorly developed mastoid cavity, cochlear dysplasia (Fig. 1A), hypoplastic semicircular canals, ossicular chain malformation, and sigmoid sinus engorgement (Fig. 1B). MRI revealed a narrow internal auditory canal and a hypoplastic cochlear nerve. Because of these findings, the patient was diagnosed as typical CHARGE syndrome with profound hearing loss. CI was performed under general anesthesia on the left side via a modified transcanal approach with EAC obliteration.

Patient has provided informed consent for publication of the case. The Ethical committee approval was acquired from the institutional ethical review board of Taichung Tzu Chi Hospital (IRB number: REC108-32).

## 3 Methods

**Figure 2:**
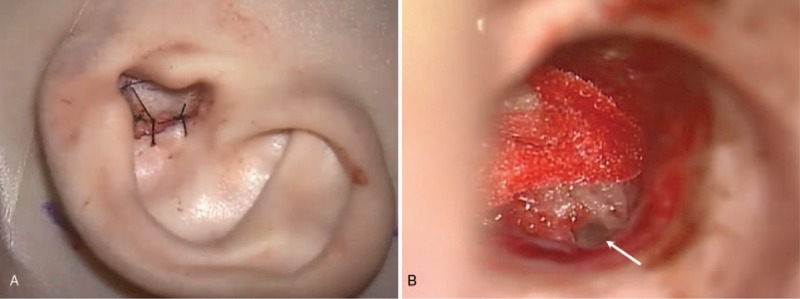
Excision of the tragal cartilage undermining of the conchal skin followed by EAC closure. Identification and opening of the round window (arrow).

**Figure 3:**
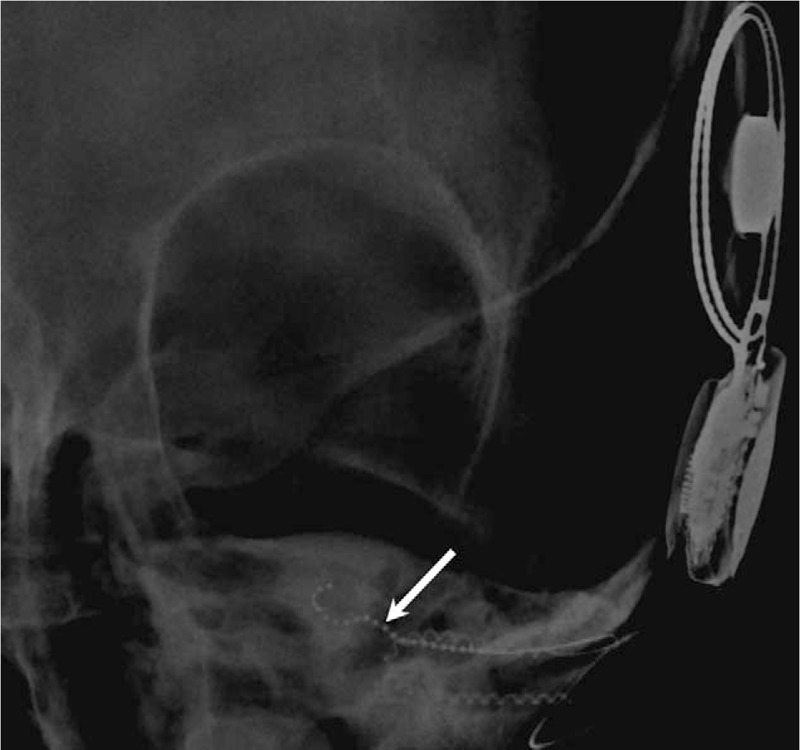
Insertion of only 10 electrodes was possible (arrow) because of her dysplastic cochlea.

The modified transcanal approach:

(1) Elevate the tragal flap by excising the tragal cartilage, then incise and undermine the conchal skin. Suture the tragal flap to the conchal skin (Fig. 2A).
(2) Create a postauricular incision to access the external auditory canal. Remove all remaining canal skin, including the tympanic membrane, followed by canalplasty.
(3) Use oto-endoscope for better visualization of the round window and middle ear structures and facial nerve monitoring to map out the course of the nerve. (Fig. 2B)
(4) Drill a cable groove in the EAC and a device well at the skull.
(5) Identify and open the round window. Insert the electrode into the scala tympani as deep as possible. In this case, only 10 electrodes were inserted because of her dysplastic cochlea (Fig. 3).
(6) Secure the cable at the EAC and the receiver-stimulator device at the skull. Close the wound layer by layer.
(7) Perform neural telemetry immediately to ensure correct electrode placement.

By using the modified transcanal approach with EAC obliteration, CI was successfully done in this patient with CHARGE syndrome. The patient had transient facial palsy (House Brackmann Grade II) postoperatively which completely resolved after 1 month. Aided threshold decreased to approximately 25 dB. The modified transcanal approach is a reasonable option for CI in CHARGE syndrome.

## 4 Discussion

CI in CHARGE syndrome confers a high rate of complications^[8,9]^ According to previous reports, CHARGE syndrome has the following anatomic defect rate: 38% to 81% had cochlea dysplasia, 28% to 81% had aberrant facial nerve course, and 77% to 93% had middle ear malformation.^[5,10]^ All of these deformities may result in a technically challenging and high risk surgery.

Several approaches to CI are available, including the facial recess approach,^[11]^ the endomeatal approach,^[12]^ and the suprameatal approach.^[13]^ These approaches naturally present various complications, including cerebrospinal fluid leak, infection, frequent facial nerve injury, massive otorrhea, cable extrusion, and cholesteatoma formation.^[14]^ For certain cases, like undeveloped mastoid, aberrant facial nerve and engorged sigmoid sinus, the transcanal approach has several advantages: it allows easier identification of the round window, obviates the need for mastoidectomy,^[15]^ and decreases the possibility of sigmoid sinus injury by bypassing the mastoid.

Obliterating the external auditory canal prevents infection and creates a cosmetically pleasing wound. External auditory canal widening yields a better visualization of major structures and easier electrode insertion. Complete removal of EAC skin, including the tympanic membrane and residual epithelium, can prevent cholesteatoma formation in the future. The use of the transcanal approach has been previously documented.^[16]^ We combined the modified transcanal approach with oto-endoscopy to optimize surgical access and exposure. Oto-endoscopy facilitates anatomic orientation and landmark identification. Moreover, facial nerve monitoring allows the surgeon to follow the course of the facial nerve and ensure its integrity during the procedure. In this case, only 10 electrodes were inserted because of her dysplastic cochlea. In the future, the use of computed tomography simulation may be considered to estimate the depth of insertion of the electrodes preoperatively and to decrease the risk of facial nerve injury.

There is no specific limitation of this technique. The surgical time was 192 minutes which is close to another report with an average of 186 minutes.^[16]^ It was still longer than the frequently used facial recess approach. Closing EAC and unfamiliarity with the approach may prolong the operating time. The possible complications of the modified transcanal approach could be cholesteatoma formation resulting from residual EAC epithelium. Therefore, complete removal of EAC epithelium is crucial before obliteration.

## 5 Conclusion

The modified transcanal approach with EAC obliteration is a feasible and safe CI technique in CHARGE syndrome. It offers a direct visualization of the round window, thereby circumventing the poorly developed mastoid.

## Author contributions

**Conceptualization:** Cheng-Yu Hsieh, Chuan-Hung Sun, Wei-Lin Lin, Giselle L. Gotamco, Chuan-Jen Hsu, Hung-Pin Wu.

**Investigation:** Wei-Lin Lin.

**Supervision:** Chuan-Jen Hsu, Hung-Pin Wu.

**Validation:** Chuan-Hung Sun, Wei-Lin Lin, Hung-Pin Wu.

**Visualization:** Cheng-Yu Hsieh.

**Writing – original draft:** Cheng-Yu Hsieh.

**Writing – review & editing:** Cheng-Yu Hsieh, Chuan-Hung Sun, Giselle L. Gotamco.

Cheng-Yu Hsieh orcid: 0000-0002-2588-8881.
